# How reticulated are species?

**DOI:** 10.1002/bies.201500149

**Published:** 2015-12-28

**Authors:** James Mallet, Nora Besansky, Matthew W. Hahn

**Affiliations:** ^1^ Department of Organismic and Evolutionary BiologyHarvard UniversityCambridgeMAUSA; ^2^ Department of Genetics, Evolution and EnvironmentUniversity College LondonLondonUK; ^3^ Department of Biological Sciences and Eck Institute for Global HealthUniversity of Notre DameNotre DameINUSA; ^4^ Department of Biology and School of Informatics and ComputingIndiana UniversityBloomingtonINUSA

**Keywords:** admixture, homoplasy, introgression, phylogenetic discordance, speciation, species concepts, tree of life

## Abstract

Many groups of closely related species have reticulate phylogenies. Recent genomic analyses are showing this in many insects and vertebrates, as well as in microbes and plants. In microbes, lateral gene transfer is the dominant process that spoils strictly tree‐like phylogenies, but in multicellular eukaryotes hybridization and introgression among related species is probably more important. Because many species, including the ancestors of ancient major lineages, seem to evolve rapidly in adaptive radiations, some sexual compatibility may exist among them. Introgression and reticulation can thereby affect all parts of the tree of life, not just the recent species at the tips. Our understanding of adaptive evolution, speciation, phylogenetics, and comparative biology must adapt to these mostly recent findings. Introgression has important practical implications as well, not least for the management of genetically modified organisms in pest and disease control.

## Introduction

Not so long ago, analysis of microbial 16S ribosomal RNA sequences led to a revolutionary new “Universal Tree of Life,” consisting of three monophyletic domains, here referred to as the Bacteria, the Archaea, and the Eukarya or eukaryotes 1, 2. Yet almost as soon as the new system was established, this tidy tree picture was threatened: sequencing of more microbial genes and then whole genomes quickly led to an understanding of the importance of horizontal or lateral gene transfer, the incorporation of foreign genes into the genome. Some of the major transitions in evolution were clearly due to lateral transfer: the eukaryotes were formed by endosymbiosis of α‐proteobacteria with Archaea to form the eukaryotes. Later, endosymbiosis of cyanobacteria with eukaryotes led to green algae and plants. Many other gene transfers together with multiple other endosymbioses have been inferred. Microbiologists began to argue that the “tree” of life was more like a web or network than a tree 3, 4, 5.

Today, whole genome sequencing is providing unprecedented phylogenetic information about whole groups of eukaryotes 6, 7, 8, 9, 10, 11, 12, 13, 14. Here we review genomic evidence suggesting that reticulate evolution may have considerable impact in multicellular eukaryotes as well as microbes. Reproductively isolated species and bifurcating phylogenies have become an important basis for our understanding of evolution; now this bedrock seems threatened. As an ideal, species are often taken to be evolutionarily independent populations that are reproductively isolated from other such species, for example in the “biological species concept,” although it was always known that hybridization does occur 15. Reticulate evolution in plants has long been recognized 16, but recent genomic evidence from animals suggest that reticulation might be much more common than anticipated 17, 18. Given abundant new data, it is time to enquire whether a major shift in our understanding of species, speciation, and phylogenetics is taking place.

## Prokaryotes: Is there a universal tree of life?

Tree‐like relationships among species arise because the genome evolves within cells. When a cell divides, copies of the same genome are found in each daughter cell. Ultimately, after populations of organisms diverge or “speciate,” evolution along each branch will leave genomic signals of that branching event in daughter lineages. Sex and recombination can obscure this picture, but in both Bacteria and Archaea sex (in the eukaryote‐like sense of homologous gene exchange) is mostly a transaction between closely related individuals, mostly within the same populations or “species” 19, 20, 21, 22. Eukaryotes are similar 23, 24. Lateral transfer involving non‐homologous exchange, on the other hand, will lead to more wide‐ranging phylogenetic discordance. In prokaryotes, both sex and lateral transfer involve relatively few genes at a time or even if more extensive, usually much less than 50% of the genome. Nevertheless, multiple exchanges may take place, and very large fractions of the genome might eventually be exchanged with other lineages or species over long periods. If so, it is possible that the signals of the organismic genealogy (the original “tree of cells”) in the genome will be obliterated by multiple phylogenetic signals from sex and lateral transfer.

Before assessing new genomic evidence for phylogenetic discordance in multicellular eukaryotes, it is worth reviewing the controversy raging about the microbial “Tree of Life” over the last few decades. Carl Woese 25 argued that in spite of considerable lateral transfer, there is “a genealogy‐defining core of genes whose common history dates back to the root of the universal tree.” Woese suggested that the acquisition of sufficient co‐adaptation among these key genes caused life to reach a “Darwinian threshold,” which permitted divergence into separate species and allowed us to trace the organismal history, even while lateral transfer obscures the universal tree for many other genes. According to Woese, before the Darwinian threshold was reached, divergence and speciation could not take place, and no tree of genes would allow us to trace the organismal history.

It quickly became apparent that lateral transfer does indeed swamp the signal of the Universal Tree in microbial genomes: in fact no other genes support Woese's original 16S RNA tree 26. Many microbiologists now deny a tree‐like phylogeny of microbial evolution; instead the phylogeny of life looks more like a web or a ring 3, 27, 28, 29. By excluding all genes that disagree with the Universal Tree, one can select 20–30 largely informational genes that more or less rescue the ribosomal RNA Tree 29, 30, 31. But this almost seems like cheating, and is itself obtained only by pruning out a number of clear cases of lateral transfer in even these genes. As this anyway only applies to a tiny fraction of the genome, these recent incarnations of the Universal Tree have been derided as “the tree of one per cent” 32. Around 80% of eukaryotic proteins are actually more closely related to homologs in the Bacteria than in the Archaea; the Universal Tree's closer archaeal‐eukaryote affinity is reflected in only about 15% of eukaryote proteins 28, 32, including those used by Ciccarelli et al. 30. Because of concerns such as these, the existence of species and of the Universal Tree in microbes has been dismissed as a “myth” in the prokaryote literature 33. Whether species or the Universal Tree exist in prokaryotes has become almost a philosophical rather than a biological issue 29, but it does seem clear that most of the original Universal Tree, whether identifiable or not, is located on the far side of what Woese originally intended by the Darwinian Threshold.

## What causes phylogenetic incongruence in eukaryotes?

Findings of promiscuous gene exchange among prokaryotes have usually been contrasted with supposedly well‐behaved trees in eukaryotes 33, 34. Eukaryote genomes originated when an archaeal cell acquired many bacterial genes, in part but certainly not only associated with the bacterial endosymbiotic origins of mitochondria and chloroplasts 35. Eukaryotes also invented meiosis, which allows recombination of whole genomes. In multicellular eukaryotes, reproduction itself often involves meiosis. This innovation effectively destroys the tree‐like signal in an organismal (“tree of cells”) phylogeny. In every meiosis recombinant haploid genomes from two successful, independent cells are thrown together to form diploid zygotes, before the sum of the genetic material is haphazardly and approximately equally recombined into haploid daughter cells. A “tree of cells” justification for the eukaryote Tree of Life is no longer possible.

While tree‐like patterns are readily discernible in eukaryote phylogenies, we here highlight recent evidence suggesting that a number of regions of the eukaryotic tree show similar pathologies to those found in prokaryotes. This raises doubt about the eukaryotic Tree of Life as a whole. Apart from phylogenetic estimation error and homoplasy, there are three main causes of phylogenetic incongruence: lateral gene transfer, incomplete lineage sorting, and introgression.

### Lateral transfer

In Eukaryotes, lateral or horizontal gene transfer is widespread, but is usually thought to be rare compared to that in prokaryotes 8, 36, 37. It seems to be associated mainly with single‐celled eukaryotes (the “protists”), especially those that engulf their food, or in multicellular organisms with parasites in close cellular contact with their hosts. Eukaryotes clearly seem to have acquired important genes via lateral transfer from both mitochondrial and chloroplast endosymbionts, but transfers also originate from other endosymbionts, parasites, and close associates 35. Lateral processes in eukaryotes, in contrast to other possible causes of reticulation, may transfer genes between distantly related species, but typically involve relatively few genes at a time, as in prokaryotes. Lateral transfer is common in some multicellular groups 36, such as bdelloid rotifers, which, interestingly, lack meiotic sex 38, 39. Horizontal gene transfer in the mitochondrial genomes of plants and yeasts is also widespread 40. However, horizontal transfer is probably not an overriding factor in the evolution of the nuclear protein coding genes of most multicellular eukaryotes, unlike those of prokaryotes.

In contrast to the genes, eukaryotic genomes often consist largely of non‐coding DNA, and 30–60% of this consists of recognizable mobile elements 41, 42. Intergenic and intronic DNA is thought to originate largely via active or inactivated mobile genetic elements 43, 44, 45, most of which are thought to enter lineages via lateral transfer 46. Mobile elements are particularly likely to be important in the evolution and spread of regulatory elements. Nonetheless, the introduction of new mobile elements via lateral transfer is rare, and the lifespan of active proliferation via transposition is cut short by relatively rapid loss, inactivation and sequence degradation in the host genome 46.

### Incomplete lineage sorting

The two main causes of gene tree – species tree discordance, at least for protein‐coding genes in closely related groups of eukaryotes, are incomplete lineage sorting and introgression. Incomplete lineage sorting occurs when polymorphisms persist between speciation events, so that the actual (true) genealogical relationship of a gene or genome region differs from the true species branching pattern. As an example of incomplete lineage sorting, around 15% of human genes are more closely related to homologs in gorillas than to those in our true sister lineage, the chimpanzees, while another 15% of genes group gorilla and chimpanzee. This is expected from what we know about the ancestral effective population sizes of these species and the short time between human‐gorilla and human‐chimpanzee speciation events 47, 48.

In some cases, incomplete lineage sorting occurs as a result of balancing selection maintaining polymorphisms: when speciation occurs, both daughter species may maintain the same “trans‐specific” polymorphisms, even though with recombination, the signal of ancestral origin may erode over time 49. Good examples of shared polymorphisms between humans and apes are MHC 50 and ABO blood group loci 51, among other genes. In the species complex including the major mosquito vector, *Anopheles gambiae*, a very large chromosomal inversion, 2La (22 Mb in length, 8.5% of the total genome size) is maintained as a balanced polymorphism that has persisted across several speciation events 18.

Unlike lateral transfer and introgression, however, discordance created by incomplete lineage sorting does not imply phylogenetic reticulation at the level of species. It merely muddles the genomic signal of what might be a truly bifurcating phylogeny. In some trees with four or more taxa and rapid successive speciation events (the “anomaly zone” of phylogenetics), the species tree estimated from the gene trees has been shown to converge on an incorrect but highly significant solution 52, 53. In spite of this “tyranny of the majority” in phylogenetic analysis, a coalescent‐based analysis should nonetheless be able to retrieve the true bifurcation signal in spite of the confused gene tree signal 54, 55.

### Introgression and reticulated evolution

The third source of phylogenetic incongruence, introgression, occurs when hybrids backcross and transfer genetic material between species. Hybridization may occur without strongly affecting the genomes of recipient populations if strongly resisted by selection, but genomic admixture results if the introgressed alleles are established.

Hybridization between related eukaryote species does occur reasonably frequently in nature; it is known to affect around 25% of the species of flowering plants and about 10% of animals 56, 57, 58. The fraction of hybrids in natural populations, nevertheless, is usually low: natural interspecific hybridization rates in animals are typically 0.1% or less per generation in any species 57, 59. Per generation hybridization rates can be much higher in some populations of plants and animals, where it reaches several per cent, for example in some oaks (*Quercus*), Darwin's finches, and some cases in *Heliconius* butterflies 60, 61, 62, 63; but these are probably exceptional. While some hybrids are sterile, a substantial fraction of such hybrids are at least partly fertile, leading to observed cases of backcrossing and introgression. It is important to realize that hybridization and introgression may occur among non‐sister species as well as between sister species, especially during rapid adaptive radiations.

Closely related species hybridize more readily than more distant species 64. The decline of natural hybridization rates with genetic distance, while noisy, may be very roughly approximated as exponential 59, mirroring the noisy decline of compatibility in meta‐analyses of transformation experiments in prokaryotes and laboratory crosses in animals and plants 19, 20, 21, 22, 23, 64, 65, 66, 67. Thus, introgression tends to generate phylogenetic discordance mainly among closely related groups of species, unlike lateral gene transfer. This is a major difference between reticulate evolution in prokaryotes and eukaryotes: while lateral gene transfer weaves lineages together across disparate parts of a tree, introgression merely results in tangled knots on a local scale. Nonetheless, introgression has potentially important effects throughout the tree of life by obscuring relationships among lineages that diversified rapidly at any time, not just in those that did so recently.

Introgression was well known before the advent of genetic markers or genomics, and was long believed an important catalyst for adaptive evolutionary change in plants 16. Introgression was thus familiar by the 1960s, but ideas of “coadapted gene complexes,” and “the unity of the genotype” associated with the biological species concept led to a belief that hybridization had little importance in animals, at least. When hybridization did occur, it was often assumed to be unnatural and was attributed to environmental changes wrought by humans 68. Because hybrids are mostly unfit, it was assumed that introgression among animal species very rarely had any long‐term evolutionary impact 15.

With the potential for introgression, not only will individual gene trees tell different stories, but the actual organismal branching pattern between species will be reticulate, rather than strictly bifurcating. The true phylogeny may be approximately tree‐like if introgression is rare and affects only a very small fraction of the genome, but will not be tree‐like if introgression is common. However, the importance of introgression is only now becoming apparent with rapid genome sequencing.

## Gene transfer is important in eukaryote genomes

### The extent of introgression across the eukaryote tree

As we have seen, meiotic fertility has an increasing tendency to fail with genetic distance, but failure is often not complete in the closest hybrids. For this reason, introgression, which requires some fertile hybrid offspring, is most likely to occur among closely related species. Hybridization between sister species will not usually affect the species tree topology, but will make the apparent divergence time between the species appear more recent 69. However, if two widely distributed species interact in populations where they overlap, it may be possible that individual populations become on average more closely related locally to a sister species than to more distant conspecific populations. In contrast, hybridization and introgression among non‐sister lineages can readily distort the species tree topology. If introgression between non‐sister lineages is widespread across the genome, it may be very hard to retrieve the true bifurcation history of the species. This is because a unitary history of the genome may not exist; if inferred from multiple loci or whole genomes, this species tree may be meaningless or misleading. Here we discuss several recent examples from multicellular eukaryotes where this may have occurred. Interestingly, most of these examples come from rapid species radiations; these are exactly the cases in which closely related but non‐sister species may be hybridizing.

**Figure 1:**
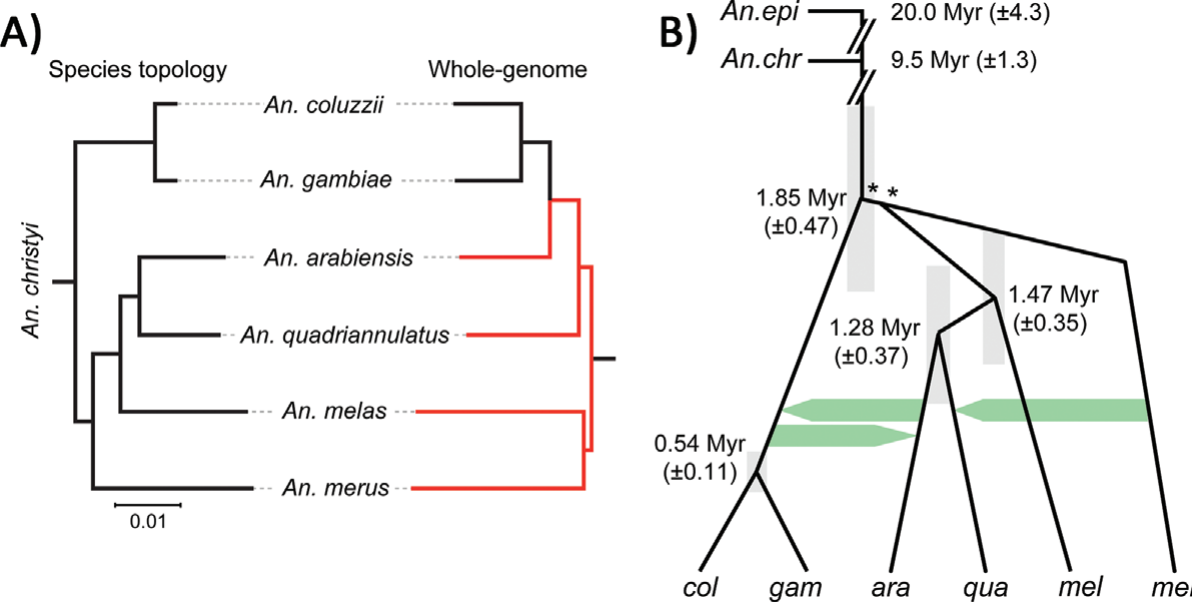
**A:** “Whole genome” versus “species” tree topologies of the *Anopheles gambiae* complex in Africa. **B:** The tree based on the X chromosome only, showing introgression events and estimated node divergence times. The average phylogeny of the whole genome is distorted by autosomal introgression between *A. gambiae + coluzzii* and *A. arabiensis*, but this was prevented on the X chromosome by X‐linked hybrid incompatibilities and multiple overlapping inversions that prevent recombination. Modified and reprinted from 18 with permission from AAAS.

The group of eight African mosquito species known as the *A. gambiae* complex radiated within the last 2 million years 18. Species distributions overlap extensively, and in areas of sympatry hybrids have been recorded at rates of ∼0.02–0.75% 70, 71. Despite F1 hybrid male sterility in most cases, introgression is plausible through the backcrossing of vigorous and fertile F1 hybrid females. When genomes of multiple members of the *A. gambiae* complex were sequenced and compared, the inferred species tree was evident in only 2% of the genome, mainly on the X chromosome, whereas the majority tree in the rest of the genome yields a completely contradictory tree 18. While some of these differences are due to incomplete lineage sorting, much of this discordance is due to introgression between two non‐sister species (Fig. 1). This is particularly clear for the 2La inversion mentioned above, which is inferred to have been polymorphic in the ancestor of the complex, but is affected by three losses of 2L+ and one of 2La, as well as one fairly recent (1 Mya) introgression of 2La from *A. gambiae* to *A. arabiensis* 18. Introgression is on‐going, and is an excellent explanation for the phylogenetic discordance, because wild hybrids and backcrosses between the latter two species are ∼0.22% of the individuals captured in sympatry 71, 72. In deciding between conflicting topologies, the species tree was inferred from regions of the genome with the deepest coalescence times between species 18. If this information had not been available, or if introgression had been even more complex, it would have been hard to infer the species tree at all.

In *Heliconius* butterflies, the “*melpomene*‐silvaniform” clade consists of around 15 species. Most of these are “good” species that co‐occur over large sympatric regions, and are somewhat interfertile with other members of the clade. However, rare hybrids and backcrosses are known from the wild and in captivity across this whole group, suggesting the possibility that a slow trickle of introgression is constantly occurring among the largely sympatric species in the group 59. This suggestion has now been confirmed: because of introgression, a local population of *H. melpomene* can be more closely related to the locally overlapping population of its sister *H. cydno* than it is to conspecifics at over 40% of the genome 17, 73.

Rapid radiations such as these tend to produce many closely related species that may be partially interfertile. For example, per generation hybridization rates among closely related species of Darwin's finches can be as much as 6%, with high fertility of hybrids. The Darwin's finches began to diversify on the Cocos and Galapagos Islands less than 1 million years ago, and there is strong genomic evidence for past and continuing introgression across almost the entire group 74. Other vertebrate groups such as African lake cichlids, *Xiphophorus* fishes, horses, and even hominins show similar phylogenetic discordance inferred to be due to introgression 75, 76, 77, 78.

Much deeper evidence of reticulate evolutionary patterns also exists. For example, there is considerable phylogenetic discordance at the base of the Neoaves, or modern birds 79, 80, 81. In fact *none* of the thousands of individual gene trees support the various conflicting estimates of the species tree 79, 81. Trees built from indels and stable mobile element insertions (which are less prone to homoplasy than nucleotide or amino acid substitutions) show similar conflict, suggesting that the gene tree discordance is real, rather than due to phylogenetic error 79. The authors of these papers argued that the tangle at the base of this ancient radiation was due to incomplete lineage sorting, but did not address the possibility of introgression. Yet introgression seems a likely additional cause: around 9% of today's bird species are known to hybridize in the wild 56, and birds retain some hybrid compatibility with congeners for ∼10 My after speciation 65. After the demise of the dinosaurs, the early Neoaves had few competitors, and it is not unlikely that the first species in today's lineages were able to hybridize with one another during their global ecological diversification, much as the Darwin's finches do today on the Galapagos Islands 74. An explanation for the strong signals of discordance at the base of the Neoaves 79, 81 may therefore lie partly in gene flow among the lineages after they diversified. Given that other major groups, such as the placental mammals 13, or the animals as a whole 82, appear also to have evolved in rapid radiations, it seems likely that our persistent problems with estimation of trees for the deepest branches of these radiations is due to historical introgression as well as incomplete lineage sorting during their initial diversification.

### Is introgression adaptive?

**Figure 2:**
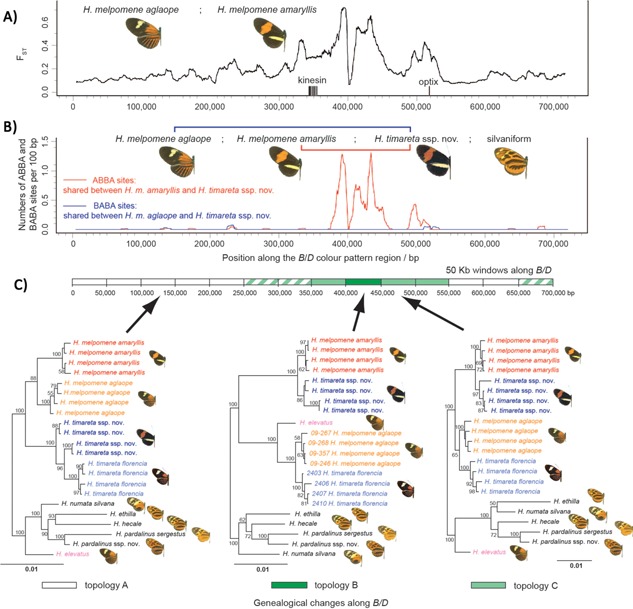
Phylogenetic discordance *B*/*D* mimicry region of *Heliconius* genomes. **A:** *F*_ST_ plot shows divergent *optix* regulatory region determining mimicry differences between geographic races within *H. melpomene*. Mimicry has been shown to have very strong adaptive value in *Heliconius*. **B:** The same region shows a strong excess of ABBA phylogenetic sites over BABA sites, implicating introgression between *H. melpomene* and *H. timareta*. **C:** Furthermore, the non‐sister species *H. elevatus* shows a phylogenetic topology indicating introgression of the rayed mimicry pattern from the *melpomene*‐*timareta* clade in the same genomic region. Modified and reprinted with permission from 17.

Phylogenetic or genealogical studies of the extent of introgression across the genome do not, however, reveal whether the process is largely neutral or whether it is aided by a selective advantage on the new genomic background. The relative importance of selection in introgression across the genome is still not known, and is an area of active research 83, but many introgression events are now known to have involved adaptation. A number of transfers of mimicry‐determining loci have been documented in *Heliconius* butterflies (Fig. 2A and B), and in *Anopheles* the many cases of insecticide resistance alleles crossing species boundaries (see below) and the existence of balancing selection at the 2La inversion make it rather hard to believe that selection is only rarely involved in introgression.

Adaptive introgression may also introduce adaptive combinations that lead to new species, or hybrid speciation 84, 85. Plant examples have long been known 16, 85, but animal examples are no longer rare. For example, the *Heliconius pardalinus*‐like ancestor of *H. elevatus* seems to have recently acquired the majority of its defensive color pattern mimicry from *H. melpomene* (Fig. 2C), subsequently proving able to coexist in sympatry with both parents 17. That case remains to be fully worked out, but similar cases have been put forward for cichlid fish, monkeyflowers, and other hybridizing adaptive radiations 86, 87. In one case, the beginnings of the process have been observed in “real time”: a new hybrid finch species that breeds strictly endogamously has now been followed on a Galapagos island for seven generations since its formation via initial hybridization events in the early 1980s 88.

## Introgression challenges notions of species and phylogeny

### The meaning of species and speciation

We are thus confronted by extraordinary levels of introgression found in the genomes of rapidly radiating species (such as *Anopheles*, *Heliconius*, and Darwin's finches). Yet these taxa are currently readily identifiable to species using morphology or genetics: none of us doubt that the species is a useful rank, at least in multicellular eukaryotes. We recognize these taxa as species not because of reproductive isolation per se, nor because they represent phylogenetic branching events, but because of the simpler observation that hybrids and intermediates between the clusters we call species 89 are rare. While most of the introgression that has resulted in reticulate relationships occurred in the past – and may or may not be ongoing – these results suggest that species are like the Ship of Theseus in philosophy, which can progressively but almost completely be rebuilt with new wood, and yet remain the same ship. We do not yet know how common these effects are among genomes of other eukaryotes, but the recent discoveries in mammals, birds, fish, insects, plants, and fungi suggest that they may be widespread throughout the eukaryotic Tree of Life.

### The “true phylogeny” versus the species tree

**Figure 3:**
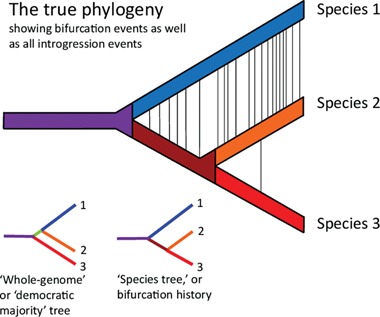
A simple case where introgression can distort the history of species and speciation. By “the true phylogeny,” in this paper, we mean the totality of true histories of every part of the genome. This is not readily depicted: our simplified cartoon of the true phylogeny network above indicates abundant introgression between species 1 and 2 after their bifurcation, but little between sister species 2 and 3. It does not, however, show which gene travels in which direction and when, all of which is surely important information about the “true phylogeny” as well. If introgression is extensive, the whole genome tree (bottom left) may indicate an incorrect bifurcation history, as well as ancestral species that never existed (such as the apparent ancestor of 1 and 2 in the diagram). The true bifurcation history of species is shown bottom right.

In introgressing species, different gene trees vary in the story they tell about their genealogical history. The true phylogeny will trace the disparate histories of every gene, and cannot readily be represented on a page, certainly not as a single tree. Yet we propose that there may still be a true bifurcating tree of species out there (Fig. 3), in spite of the background chaos of gene trees. Only if species fuse either wholly or in some geographic region to become a single cluster (e.g. in sticklebacks 90 or in hybrid speciation), does the species phylogeny itself become reticulate under this view.

Possible alternatives to the species tree is some consensus of gene trees, or perhaps the tree based on the “democratic majority” of the genome 91. Obtaining the maximum likelihood or most probable species tree from a series of genes is in fact the aim of many phylogenetic and phylogenomic studies, at least among eukaryote systematists 92, 93. This program assumes that the true species tree is more likely to emerge via analysis of larger fractions of the genome. Under the viewpoint proposed here, this is not necessarily true if there is abundant introgression (Fig. 3). For example, as shown above, the single most common tree inferred from whole genomes of the *Anopheles gambiae* complex in Africa gives an incorrect rendering of the group's history 18 (Fig. 1).

Historical introgression events in taxa such as *Anopheles* have been inferred to affect the majority of the genome, even though natural hybrids are relatively rare among the contemporary species (see above for rates of hybridization). Nonetheless, hybridization can introduce variation at rates much higher than mutation, so that significant levels of genomic replacement may accrue over long periods, even at the low hybridization rates known in *Anopheles* today. Similar results also apply in some *Heliconius* species. If we wish the species tree to be determined by the democratic opinion of the genes, we are therefore forced to accept a peculiar species definition that perhaps applies only to terminal taxa, rather than the original bifurcating ancestors, because the branches of the tree change their species identity whenever accumulation of introgressed regions flips the democratic majority of the genes to another topology. It is perhaps defensible to argue that the “democratic opinion” tree is more predictive of the origins of the genes, though it is marred by potential inferences of ancestral species (pale green) that never existed (Fig. 3). We instead favor the idea that the species tree is the bifurcation history (Fig. 3). This we would argue is closer to what we mean by the speciation history, in spite of the difficulty of its discovery, and acknowledging a lowered expectation of its predictiveness for the histories of its component genes.

### Are species incompatible?

Another conclusion that arises from these findings is that large fractions of different species’ genomes may in fact be compatible. The genomic distribution of “intrinsic” incompatibilities (such as “Dobzhansky‐Muller incompatibilities” 94, 95) is poorly known except in a few species 96. In *Saccharomyces* yeasts, it is possible to replace whole chromosomes with little effect on viability, while in *Drosophila* many hybrid sterility loci seem scattered very widely across the genome 97, 98, 99, 100, 101. It is possible that the situation in *Drosophila* is unusual, perhaps a result of “faster male” sexual selection that leads to genome‐wide effects on male hybrid sterility 102, 103. Even though incompatibility loci have been mapped in crosses between *A. gambiae* and *A. arabiensis* 104, genomic evidence for very widespread homologous replacement between species in the autosomes of *Anopheles* and *Heliconius* 18, 73 suggests either that incompatibilities were not very common in those genomes, or that some introgressed alleles are advantageous enough to overcome initial incompatibility. Although autosomal genes introgress readily in both groups, the preponderance of “species tree” genealogies in the sex chromosome in the *Anopheles gambiae* complex 18 is likely due to multiple overlapping inversions that differ between *A. gambiae* + *coluzzii* and *A. arabiensis*. These inversions suppress recombination and so inhibit introgression of small chromosomal fragments on this chromosome. If adaptive alleles are widely available to introgress, determining the number and effect of incompatibilities will not be adequate to assess the potential for introgression between species: we will also need to know the number and selective effects of these variants.

As far as is known, classical lateral transfer from distantly related species is not a major recent source of phylogenetic incongruence in multicellular eukaryotes, and most of the phylogenetic reticulation we observe is due to homologous exchange via hybridization. The selective advantages of sex within species remain contentious, but sex surely optimizes some balance between benefits and costs of recombination 105, 106. Typically, hybridizing with another species is viewed as “the grossest blunder in sexual preference,” and mate choice (reinforcement) is expected to evolve to limit hybridization among sympatric species 107. However, given that hybridization does still occur, and sometimes leads to beneficial effects, we should now perhaps broaden our view of sex across the species boundary, where the same cost/benefit function is confronted by individuals seeking sexual partners, albeit with different parameter values. If outcrossing within and between species is regulated by the same cost/benefit equation, a sexual selection process similar to reinforcement should apply to interactions within as well as between species.

### Practical implications of introgression

The prevalence of laterally transferred antibiotic resistance genes among bacterial species is a well‐known problem for human health 108, 109. Similar problems might therefore be expected to result from introgression or lateral transfer among related eukaryotic pest and disease species. The African malaria‐carrying mosquitoes provide some worrying examples. For example, rates of hybridization between *Anopheles gambiae* and *A. arabiensis* are only ∼0.22% per generation 71. However, because this introduces foreign alleles at a rate far higher than mutation, there are persistent concerns that insecticide resistance evolution in one species may lead to the rapid spread of that resistance to others via introgression 72. Multiple cases of introgression of alleles encoding both organophosphate and pyrethroid insecticide resistance are certainly known between the sister species *A. gambiae* and *A. coluzzii* 110, 111, 112, 113; these two are known to hybridize and backcross much more frequently 63 than do *A. gambiae* with *A. arabiensis*. Similarly, large sibling species complexes of the black fly genus *Simulium* transmit river blindness in Africa and tropical America, and may also exchange genes. Among sympatric species of the African *S. damnosum* complex, hybridization rates may reach 0.1% per generation. Introgression is thought likely to explain the rapid spread of insecticide resistance among multiple *Simulium* species in Africa 114. The same problem occurs even in vertebrate pests: a genomic region containing a rodenticide resistance allele spread via introgression between two partially interfertile mouse species in Western Europe 115.

Recent advances in genetics and genetic engineering are revolutionizing pest control, allowing for “designer organisms” in agriculture and human health. Several major transgenic crops, especially those expressing herbicide or insect resistance, have been released in many countries. At the same time, new molecular marker and genomic analyses let us gather evidence on gene flow between crops and wild relatives for the first time. The results are clear: introgression does occur, and weedy relatives are acquiring novel genetic variation from crops, including transgenes that are liable to make these weeds more noxious 116.

The use of transgenic organisms is more advanced in agriculture than in human health. However, a variety of genetic control measures of vectors have been suggested and in some cases are being used to engineer disease vector populations 117. For example, infection of *Aedes* mosquitoes by *Wolbachia* causes refractoriness to dengue virus proliferation 118, while *Wolbachia*‐infected *Anopheles* mosquitoes show reduced *Plasmodium* infection 119. In addition, there is the possibility of manipulating the genetics of mosquito innate immunity in order to reduce their efficiency as a vector 120. Of these, probably the most successfully used cases so far are a number of releases of *Wolbachia*‐infected *Aedes aegypti* to control dengue (118 www.eliminatedengue.com). As with transgenic plants, because the transmission of genetic traits requires mating, these genetic traits may “leak” to related species via introgression. This may not have negative impacts, especially in comparison to the potentially positive benefits of the engineered trait on the target species. However, given genomic evidence for introgression of many other traits, its importance should not be underemphasized when seeking regulatory approval for release of genetically modified organisms (recognizing that *Wolbachia* infection does not technically qualify as a genetic modification to most regulatory bodies).

## Conclusions and outlook

Our main conclusion is that many more species are likely exchanging genes than has been appreciated. It is not only sister species that hybridize and undergo genomic introgression: whole groups of rapidly radiating species may exchange adaptive as well as non‐adaptive genomic regions, as in *Heliconius*, *Anopheles*, cichlids, *Xiphophorus*, Darwin's finches, horses, and hominins. In fact, because hybridization between sister species does not always affect the species tree – and because introgression between sister species is more likely – it may be that estimates of introgression rates from species tree topologies alone vastly underestimate the amount of gene flow occurring in nature. For many systems we may think we are able to infer a species tree signal, but we must recognize that this signal may only be represented by a small fraction of genes.

As well as causing problems for phylogenetics, abundant introgression and incomplete lineage sorting might greatly weaken inferences in comparative analysis. When we map character traits onto the tree of a rapidly radiating group, we should be cautious. For instance, the raptorial habit is thought to be ancestral to the entire core landbirds, but today it is present in several monophyletic groups, each more closely related to birds that have apparently lost the habit 79, 80, 81. Alternatively, core landbirds may have been ancestrally non‐raptorial, and a number of raptorial traits could have could have been shared at the base of these lineages by introgression among the early species of each lineage. This is not dissimilar to what we observe in mimicry patterns in *Heliconius* or in beak morphology of Darwin's finches, among species of radiations that we see hybridizing today 17, 74. Similarly, inferences from phylogeography – such as geographic origins of rapidly radiating groups inferred from phylogenetic methods – should be affected as well. The origins of traits, and the genes that determine them can have very different histories from that of the species tree.

The authors have declared no conflicts of interest.
